# Real-world efficacy and safety of efgartigimod in patients with autoimmune encephalitis: a single-center retrospective analysiss

**DOI:** 10.3389/fimmu.2026.1963052

**Published:** 2026-10-08

**Authors:** Zhijiao Song, Lantao Liang, Meiling Zhang, Yu Zhang, Hui Bu, Yanbo Zhang

**Affiliations:** 1 Department of Neurology, The Second Hospital of Hebei Medical University, Shijiazhuang, Hebei, China; 2 Key Laboratory of Neurology of Hebei Province, The Second Hospital of Hebei Medical University, Shijiazhuang, Hebei, China; 3 Department of Encephalopathy, Hebei University of Chinese Medicine Hebei Provincial Hospital of Traditional Chinese, Shijiazhuang, Hebei, China

**Keywords:** autoimmune encephalitis, clinical efficacy, efgartigimod, neonatal Fc receptor antagonist, retrospective study

## Abstract

**Objective:**

This study was conducted to evaluate the efficacy and safety of efgartigimod (EFG), a neonatal Fc receptor (FcRn) antagonist, in the treatment of autoimmune encephalitis (AE) under real-world conditions and to provide clinical evidence for treating AE patients who respond poorly to first-line and/or second-line immunotherapy.

**Methods:**

In this study, clinical data of 14 AE patients who were treated with EFG at the Department of Neurology, The Second Hospital of Hebei Medical University between November 2023 and November 2025 were retrospectively analyzed. Treatment efficacy, disease prognosis, and adverse reactions were assessed.

**Results:**

In total, 14 AE patients were included, 11 with anti-N-methyl-D-aspartate receptor (NMDAR) encephalitis, and one each with anti-metabotropic glutamate receptor 5 (mGluR5) antibody-associated encephalitis, anti-leucine-rich glioma-inactivated protein 1 (LGI1) antibody-associated encephalopathy, and anti-IgLON family protein 5 (IgLON5) antibody-associated encephalopathy. The baseline median Modified Rankin Scale (mRS) score was 3.00 (3.00, 4.00), and the baseline median Clinical Assessment Scale for Autoimmune Encephalitis (CASE) score was 5.00 (4.25, 12.75). After EFG treatment, both mRS and CASE scores decreased significantly at all follow-up time points (Friedman test, P< 0.05). By week 12, 92.86% of patients achieved a favorable functional outcome (mRS ≤ 2). Clinical Global Impressions (CGI) scores indicated that most patients experienced clinically meaningful overall improvement, consistent with the improvements observed in the above scores. The Anti-NMDAR Encephalitis One-Year Functional Status (NEOS) score suggested that the overall prognosis of patients was satisfactory, and no relapses occurred during follow-up. Regular monitoring of peripheral blood immunoglobulin G (IgG) levels in nine patients showed a marked reduction at one week and three weeks after EFG treatment compared to baseline levels. The levels were higher at 12 weeks compared to those in the third week, although the levels remained below baseline. No patient required treatment discontinuation due to severe adverse reactions throughout the course of treatment.

**Conclusion:**

When AE patients with poor response to first-line and/or second-line immunotherapy were administered EFG treatment, they showed rapid and significant clinical improvement, favorable prognosis, and few adverse reactions. However, the long-term efficacy and safety of EFG require validation in multicenter, large-sample studies.

## 1 Introduction

Autoimmune encephalitis (AE) is a type of inflammatory response in the central nervous system (CNS) mediated by autoimmune mechanisms. It progresses rapidly, causing parenchymal brain damage and severe symptoms (1). Clinical manifestations include seizures, psychiatric and behavioral abnormalities, loss of memory, cognitive impairment, speech disorders, movement disorders, autonomic dysfunction, involuntary movements, and, in severe cases, loss of consciousness and coma (2). AE accounts for 10–20% of encephalitis cases. In 2014, AE had a prevalence of 13.7 per 100,000 individuals and an incidence of 0.8 per 100,000 person-years. The prevalence of AE is increasing, possibly attributable to improved detection of autoantibody-positive cases (3). Among the different types of AE, anti-N-methyl-D-aspartate receptor (NMDAR) encephalitis is the most common, accounting for 54–80% of AE cases (4), followed by anti-leucine-rich glioma-inactivated protein 1 (LGI1) antibody-associated encephalitis and anti-gamma-aminobutyric acid type B receptor (GABABR) antibody-associated encephalitis (4, 5). Following the discovery of anti-NMDAR encephalitis, the ease of antibody testing has improved the understanding of AE subtypes among neurologists (6). Additionally, many case reports and case series of encephalitis, encephalopathy, and syndromes associated with antibodies against cell surface antigens (CSAab) and antibodies against intracellular synaptic antigens (SyAab) have been reported in China (7–11).

The pathogenesis of AE is complex and involves aberrant activation of both humoral and cellular immunity. Depending on the location of anti-neuronal antibodies, AE can be classified into two types: antibodies against intraneuronal antigens (INAab)-associated AE and CSAab- or SyAab-associated AE (12). INAab-associated AE generally targets antigens located in the neuronal cytoplasm or nucleus; most patients with this type of AE have an underlying tumor. Moreover, this type of AE is relatively rare. It is mediated predominantly by cytotoxic T cells, leads to irreversible neurological dysfunction, and responds poorly to immunotherapy. CSAab- or SyAab-associated AE is a more common type of AE and includes anti-NMDAR encephalitis, anti-LGI1 antibody-associated encephalitis, anti-GABABR antibody-associated encephalitis, and anti-metabotropic glutamate receptor 5 (mGluR5) antibody-associated encephalitis (2). CSAab- or SyAab-associated AE causes reversible neuronal dysfunction primarily by involving the humoral immune system and generally has a favorable response to immunotherapy (13, 14).

Existing immunotherapy for AE mainly follows a step-up approach based on the clinical course of the patient. It includes first-line immunotherapy, second-line immunotherapy, long-term (maintenance) immunotherapy, escalation immunotherapy, and add-on immunotherapy (2). In the acute phase, patients are administered first-line immunotherapy, mainly involving glucocorticoids (GC), intravenous immunoglobulin (IVIG), or plasma exchange (PE), which are commonly used for treating AE patients (15–18). When first-line therapy results in unsatisfactory improvement, patients are administered second-line therapy, mainly with rituximab (RTX) (16, 19). If RTX is contraindicated, intravenous cyclophosphamide (CTX) may be used. Refractory AE patients who do not respond adequately to either first-line or second-line immunotherapy may be administered mycophenolate mofetil (MMF), azathioprine, and repeated RTX as long-term (maintenance) immunotherapy (16, 20, 21). The “Expert Consensus on the Diagnosis and Management of Autoimmune Encephalitis in China (2017 edition)” (22) defines refractory AE as AE with poor efficacy after two weeks of first-line immunotherapy. It defines relapsed AE as the reappearance of symptoms or worsening of symptoms (an increase of ≥1 point on the modified Rankin Scale [mRS]) after the patient has shown improvements or remained stable for more than two months. Some patients show poor response to conventional therapy or face long-term risks of infection and organ toxicity from immunosuppressants. These issues pose multiple therapeutic challenges and create an urgent need for more targeted and safer treatment strategies. By conducting extensive research on AE, researchers have generated many novel therapeutic agents; however, most clinical evidence remains at the level of case reports or small-sample studies, which are still in the exploratory stage. The choice of therapeutic agents and the timing of their administration are major challenges.

FcRn is widely expressed on mucosal epithelial cells of the respiratory, reproductive, and digestive tracts. It is also expressed in the placenta, mammary gland, kidney, lung, vascular endothelium, blood–brain barrier, and on some immune cells (23). The Fc region of IgG binds to FcRn in a pH-dependent manner; the binding affinity is high at endosomal pH (~6.0) but is low at physiological pH (~7.4) (24). By binding to IgG, FcRn prevents the degradation of IgG by intracellular lysosomes and transports it to the extracellular compartment under near-neutral pH conditions, prolonging its half-life and facilitating recycling. Thus, FcRn serves as an important receptor for maintaining the homeostasis of serum IgG (25). However, the pathological accumulation of pathogenic IgG is closely associated with various neuroimmunological diseases. With deeper insights into the pathogenesis of autoimmune diseases and further development of monoclonal antibody technology, FcRn antagonists have gradually gained popularity as targeted therapy for IgG-mediated neuroimmunological diseases. Efgartigimod (EFG) was approved by the U.S. Food and Drug Administration (FDA) on December 17, 2021, for treating adult patients with anti-acetylcholine receptor (AChR) antibody-positive generalized myasthenia gravis (gMG) (26). EFG shows favorable efficacy, indicating that this therapeutic approach is clinically feasible and laying the foundation for developing more efficient and precise treatment regimens.

Research on the effectiveness of FcRn antagonists in the treatment of AE is still in its early stages, and information remains limited. Data from some animal studies (27) have provided strong support for the favorable efficacy of FcRn antagonists, and several case reports and case series (28–30) have described rapid and significant clinical improvement after treatment. These findings collectively suggest that FcRn antagonists may be an effective alternative strategy when standard immunotherapy fails. However, this hypothesis needs to be validated through clinical cohort studies. Only one phase 2 clinical trial for LGI1 encephalitis (NCT04875975) is currently ongoing (31). In this study, data on demography, specific treatment regimens, disease severity, and neurological function scores were collected from AE patients (including first-episode, refractory, and relapsed cases) treated with the FcRn antagonist EFG at the Second Hospital of Hebei Medical University from November 2023 to November 2025, and the efficacy and prognosis of these AE patients were analyzed. The findings provide novel information that can help develop more efficient and precise treatment strategies, improve the quality of life of patients, and decrease disability rates.

## 2 Methods

### 2.1 Study population

This study retrospectively included 14 patients with AE who were administered EFG treatment at the Department of Neurology, The Second Hospital of Hebei Medical University between November 2023 and November 2025. All participants and their legal guardians provided written informed consent before the study was conducted. The study protocol was approved by the Ethics Committee of The Second Hospital of Hebei Medical University (Ethics Approval No.: 2025-R838). The inclusion criteria were as follows (1), Patients who were ≥ 18 years; (2) Patients who were confirmed to have AE according to the Expert Consensus on the Diagnosis and Management of Autoimmune Encephalitis in China (2022 edition) [2]; (3) Patients with poor response, relapse, or intolerance after standard immunotherapy (including but not limited to first-line therapy: GC, IVIG, PE; second-line therapy: RTX, CTX, etc.). Poor response was defined as less than a one-point improvement in the modified Rankin Scale (mRS) score at one week after at least one cycle of standard first-line immunotherapy compared to the score at baseline, or no significant relief of major clinical symptoms such as psychiatric and behavioral abnormalities, seizures, impaired consciousness, or movement disorders; (4) Patients who received at least one dose of EFG treatment with clear medication records, including timing of administration and dosing regimen (e.g., weekly dose, treatment course), and at least one efficacy and safety assessment after treatment. The exclusion criteria were as follows: (1) Patients with active, uncontrolled systemic infection at baseline, such as sepsis or active pulmonary tuberculosis; (2) Those with severe underlying diseases, e.g., organ failure, coagulation disorders, or concomitant systemic autoimmune diseases; (3) Patients for whom the records of EFG treatment were incomplete or key clinical data (e.g., baseline mRS before treatment, efficacy and safety assessments after treatment) were missing, which precluded valid analysis; (4) Patients who were pregnant or breastfeeding.

### 2.2 Data collection

Demographic baseline data were collected on sex, age, and significant past medical history. The time of first AE diagnosis, length of hospital stay, major symptoms (e.g., psychiatric and behavioral abnormalities, seizures, memory impairment, speech disorders, reduction in consciousness levels, movement disorders, central hypoventilation, etc.), and their time to resolution were recorded. The results of key ancillary investigations, including serum and cerebrospinal fluid antibody types and titers, peripheral blood total IgG, brain magnetic resonance imaging (MRI), and electroencephalogram (EEG), were collected. Before treatment, baseline scores, including the modified Rankin Scale (mRS), the Clinical Assessment Scale for Autoimmune Encephalitis (CASE), the Glasgow Coma Scale (GCS), the Clinical Global Impressions (CGI) scale, and the Anti-NMDAR Encephalitis One-Year Functional Status (NEOS) score, were recorded. The treatment regimen involved administration of 10 mg/kg EFG (a recommended dose of 1200 mg per administration for patients weighing ≥120 kg) once weekly. The specific treatment course depended on the therapeutic response of the patient. Other immunotherapeutic regimens administered before EFG, as well as the specific dose, frequency, and reasons for the discontinuation of EFG (e.g., low efficacy, adverse reactions, financial issues, insufficient clinical improvement, etc.), were recorded. Patient outcomes and prognosis were assessed through visits to the outpatient clinic and follow-up via telephone calls.

### 2.3 Evaluation of efficacy and prognosis

(1) Changes in the mRS score, CASE score, and CGI scale from baseline were evaluated at one week, two weeks, three weeks, four weeks, and 12 weeks after treatment to assess disease severity, efficacy, and adverse reactions. (2) Changes in the NEOS score and the GCS score from baseline were evaluated at one week and 12 weeks after treatment to assess patient prognosis. (3) Changes in total IgG levels in peripheral blood from baseline were evaluated at one week, three weeks, and 12 weeks after treatment. (4) The time required for the improvement of symptoms was recorded (symptom improvement was defined as the first time point at which any core clinical symptom showed improvement or the mRS score decreased by ≥1 point from baseline); (5) The proportion of patients with a favorable prognosis (defined as mRS score ≤2) and the proportion of patients with relapse were assessed.

### 2.4 Safety assessment

#### 2.4.1 Recording of adverse events

All adverse events (AEs) and serious adverse events (SAEs), including headache, diarrhea, nausea, back pain, arthralgia, allergic reactions, infusion reactions, etc., occurring during and after treatment were comprehensively collected. All infections (e.g., upper respiratory tract infection, pneumonia, urinary tract infection, bloodstream infection, etc.), including infection type, severity, time of occurrence, temporal relationship with drug administration, and outcome, were recorded. Changes in blood routine and biochemical parameters, including clinically significant abnormal changes in blood routine, liver and kidney function, electrolytes, etc., during treatment were documented. AE was defined as any unfavorable medical event occurring after the administration of the study drug, but not necessarily having a causal relationship with the study drug. SAE was defined as any medical event occurring during the clinical study that results in hospitalization or prolongation of ongoing hospitalization, causes persistent or significant disability/incapacity, is life-threatening or leads to death, or causes congenital anomaly/birth defect.

#### 2.4.2 Grading and attribution of adverse events

(1) Severity grading: Grading was performed based on The National Cancer Institute Common Terminology Criteria for Adverse Events (CTCAE) version 5.0 (Grade 1: mild; Grade 2: moderate; Grade 3: severe; Grade 4: life-threatening; Grade 5: death); (2) Causality: The relationship between the adverse event and the administration of EFG was determined based on clinical judgment.

### 2.5 Statistical analysis

(1) All statistical analyses were performed using SPSS version 27.0 software. Categorical data were expressed as counts (relative proportions) [n (%)]. For continuous variables, histograms and the Shapiro–Wilk test were used to determine whether the data followed a normal distribution. Normally distributed continuous variables, including age, length of hospital stay, time to symptom improvement, etc., were presented as the mean ± standard deviation (± S). Continuous variables that did not follow a normal distribution, including symptom duration, time from disease onset to EFG administration, frequency of administration, etc., were described using median and interquartile range [M (P25, P75)]. (2) The frequency, proportion, and median mRS score at each follow-up time point (baseline [0 week], one week, two weeks, three weeks, four weeks, and 12 weeks after treatment) were calculated, along with the proportion of patients who achieved a favorable outcome (defined as mRS score ≤2). (3) The differences in the mRS score, CASE score, and peripheral blood total IgG levels before and after treatment were compared by the Friedman test. Paired t-tests (for normally distributed data) or Wilcoxon signed-rank tests (for non-normally distributed data) were conducted to compare the differences between baseline and each post-treatment time point: mRS and CASE scores in weeks 1, 2, 3, 4, and 12; and total IgG levels in peripheral blood at one, three, and 12 weeks after treatment.

## 3 Results

### 3.1 Demographic and clinical characteristics

Based on the inclusion and exclusion criteria, 14 patients with AE who received EFG treatment between November 2023 and November 2025 were enrolled in this study, including seven patients with first-onset disease (50.50%), two patients with relapsed disease (14.29%), and five patients with refractory disease (35.71%). The median baseline mRS score was 3, and five patients (35.71%) had severe disease (mRS ≥4). This cohort had eight male patients (57.14%) and six female patients (42.86%), with an age range of 18–76 years (46.00 ± 21.27 years). Five patients (35.71%) had pre-existing underlying diseases, and four patients (28.57%) had a history of prodromal infection.

The participants included 11 cases of anti-NMDAR encephalitis (78.57%), one case of anti-mGluR5 antibody-associated encephalitis (7.14%), one case of anti-LGI1 antibody-associated encephalopathy (7.14%), and one case of anti-IgLON5 antibody-associated encephalopathy (7.14%). All participants had previously received first-line and/or second-line immunotherapy. Among them, 13 participants (92.86%) received GC, 11 participants (78.57%) received IVIG, one participant (7.14%) received PE, two participants (14.29%) received RTX, four participants (28.57%) received ofatumumab (OFA), and five participants (35.71%) received MMF (**Table 1**; **Supplementary Table S1**).

**Table 1:** Identifying information of patients with AE.

Variables	Quantity(%)/median(interquartileinterval)
Sex	
Male	8(57.14%)
Female	6(42.86%)
Age at onset, y ( $\bar{X}\pm S$)	46.00 ± 21.27
Type of disease	
Initial onset	7(50.00%)
Refractory	2(14.29%)
Relapsing	5(35.71%)
Severe patients(mRS≥4)	5(35.71%)
Antibody type	
Anti-NMDAR antibody	11(78.57%)
Anti-LGI1 antibody	1(7.14%)
Anti-mGluR5 antibody	1(7.14%)
Anti-IgLON5 antibody	1(7.14%)
History of prodromal infection	4(28.57%)
Previous treatment	
GC	13(92.86%)
IVIG	11(78.57%)
PE	1(7.14%)
RTX	2(14.29%)
OFA	4(28.57%)
MMF	5(35.71%)

It is noteworthy that after the initiation of EFG, no additional adjunctive immunotherapies were introduced for any patient, and no new immunomodulatory interventions were initiated. The only exception was that some patients who had previously received high-dose glucocorticoid pulse therapy continued to receive low-dose oral glucocorticoids as maintenance therapy. This approach minimized confounding factors derived from concomitant immunotherapies during the EFG treatment period and helped strengthen the credibility of real-world efficacy evidence for EFG.

The clinical features of the 14 participants were retrospectively analyzed (**Table 2**). The presenting symptoms included cognitive impairment in 11 cases (11/14), psychiatric symptoms in 10 cases (10/14), movement disorders in eight cases (8/14), autonomic dysfunction in seven cases (7/14), seizures, speech disorders, and impaired consciousness in six cases each (6/14), anxiety and depression in five cases (5/14), brainstem dysfunction in three cases (3/14), and sleep disorders in one case (1/14). The mean length of hospital stay was 16.50 ± 10.72 days, and the median time from disease onset to admission was 17.50 days (**Table 2**).

**Table 2:** Clinical presentation of patients with AE.

Variables	Quantity(%)/median(interquartileinterval)
Symptoms	
Cognitivedysfunction	11(78.57%)
Psychiatric symptoms	10(71.43%)
Movement disorder	8(57.14%)
Autonomic dysfunction	7(50.00%)
Seizures	6(42.86%)
Speech disorder	6(42.86%)
Disturbance of consciousness	6(42.86%)
Anxiety and depression	5(35.71%)
Brainstem dysfunction	3(21.43%)
Sleep disorder	1(7.14%)
Duration of symptoms, d	17.50(7.75,28.75)
Length of hospital stay, d ( $\bar{X}\pm S$)	16.50 ± 10.72
Time from onset to EFG administration, d	19.00(12.75,35.75)
Frequency of medication	2.50(1.00,3.75)
Time to symptom improvement, d ( $\bar{X}\pm S$)	9.62 ± 8.18
First symptom to improve	
Psychiatric symptoms	5(35.71%)
Movement disorder	2(14.29%)
Disturbance of consciousness	2(14.29%)
Seizures	1(7.14%)
Speech disorder	1(7.14%)
Muscle fasciculations	1(7.14%)
Headache	1(7.14%)

### 3.2 Accessory examination

#### 3.2.1 Lumbar puncture pressure and cerebrospinal fluid examination

All patients underwent lumbar puncture, and their cerebrospinal fluid (CSF) was examined after admission. Two patients (2/14) had high opening pressure. The mean opening pressure was 150.38 ± 67.04 mmH_2_O (normal range: 80–180 mmH_2_O). In the CSF, white blood cell count was 10.00 (2.75, 37.25) × 10^6^/L (normal range: 0–8 × 10^6^/L), glucose content was 4.00 ± 0.76 mmol/L (normal range: 2.50–4.50 mmol/L), chloride content was 123.67 ± 3.36 mmol/L (normal range: 120.00–130.00 mmol/L), and protein content was 0.51 ± 0.23 g/L (normal range: 0.15–0.45 g/L) (**Table 3**).

**Table 3:** Diagnostic tests of patients with AE (baseline).

Variables	Quantity(%)/median(interquartileinterval)
Cerebrospinal fluid parameters (baseline)	
Lumbar puncture pressure (mmH_2_O, $\bar{X}\pm S$)	150.38 ± 67.04
WBC (10^6^/L)	10.00(2.75,37.25)
Protein (g/L, $\bar{X}\pm S$)	0.51 ± 0.23
Glucose (mmol/l, $\bar{X}\pm S$)	4.00 ± 0.76
Chloride (mmol/l, $\bar{X}\pm S$)	123.67 ± 3.36
Antibody titer (baseline)	
Anti-NMDAR antibody(CSF)	10.00(10.00,10.00)
Anti-NMDAR antibody(serum)	10.00(0.00,21.00)
Anti-LGI1 antibody(CSF)	1:32
Anti-LGI1 antibody(serum)	1:100
Anti-mGluR5 antibody(CSF)	1:32
Anti-mGluR5 antibody(serum)	1:320
Anti-IgLON5 antibody(CSF)	1:32
Anti-IgLON5 antibody(serum)	1:320
Blood tests	
Elevated total IgG	6(42.86%)
MRI	
Normal	6(42.86%)
Abnormal	8(57.14%)
Frontal lobe	5(35.71%)
Parietal lobe	4(28.57%)
Insular lobe	4(28.57%)
Temporal lobe	3(21.43%)
Hippocampus	1(7.14%)
Basal ganglia	1(7.14%)
Corona radiata	1(7.14%)
Thalamus	1(7.14%)
Pons	1(7.14%)
Meninges	1(7.14%)
EEG	
Normal	4(28.57%)
Abnormal	10(71.43%)
Slow waves	9(64.29%)
Epileptiform discharges	2(14.29%)

#### 3.2.2 Antibody types and titers

Among the 14 participants, anti-NMDAR antibodies were detected in the CSF of 11 participants, with the highest titer of 1:100. Among them, seven participants also tested positive in serum, with the highest titer of 1:320, while the remaining four participants had no detectable antibodies in serum. Anti-LGI1 antibodies were detected in the serum and CSF of one patient (serum titer 1:100, CSF titer 1:32). Anti-mGluR5 antibodies were detected in the serum and CSF of one patient (serum titer 1:320, CSF titer 1:32). Anti-IgLON5 antibodies were detected in the serum and CSF of one patient (serum titer 1:32, CSF titer 1:320) (**Table 3**). Antibody testing was performed at Guangzhou KingMed Diagnostics and Oumeng Weiyi Medical Laboratory based on a cell-based assay (CBA). Suspicious results were verified by a tissue-based assay (TBA).

#### 3.2.3 Blood tests

Total IgG levels in the peripheral blood were measured in all 14 participants. At admission, six patients (6/14) had considerably higher total IgG levels, with the highest value reaching 31.4 g/L (normal range: 8.6–14.7 g/L) (**Table 3**).

#### 3.2.4 Cranial MRI

All 14 AE patients underwent cranial MRI during admission (**Figure 1**). Abnormal MRI findings were observed in eight patients (57.14%). Lesions were detected in the frontal lobe, temporal lobe, parietal lobe, insula, hippocampus, basal ganglia, corona radiata, thalamus, pons, and meninges. Lesions were the most common in the frontal lobe, occurring in five patients (35.71%). Contrast-enhanced cranial MRI was performed in four patients, and 75.00% of patients (3/4) showed contrast-enhancing lesions.

**Figure 1:**
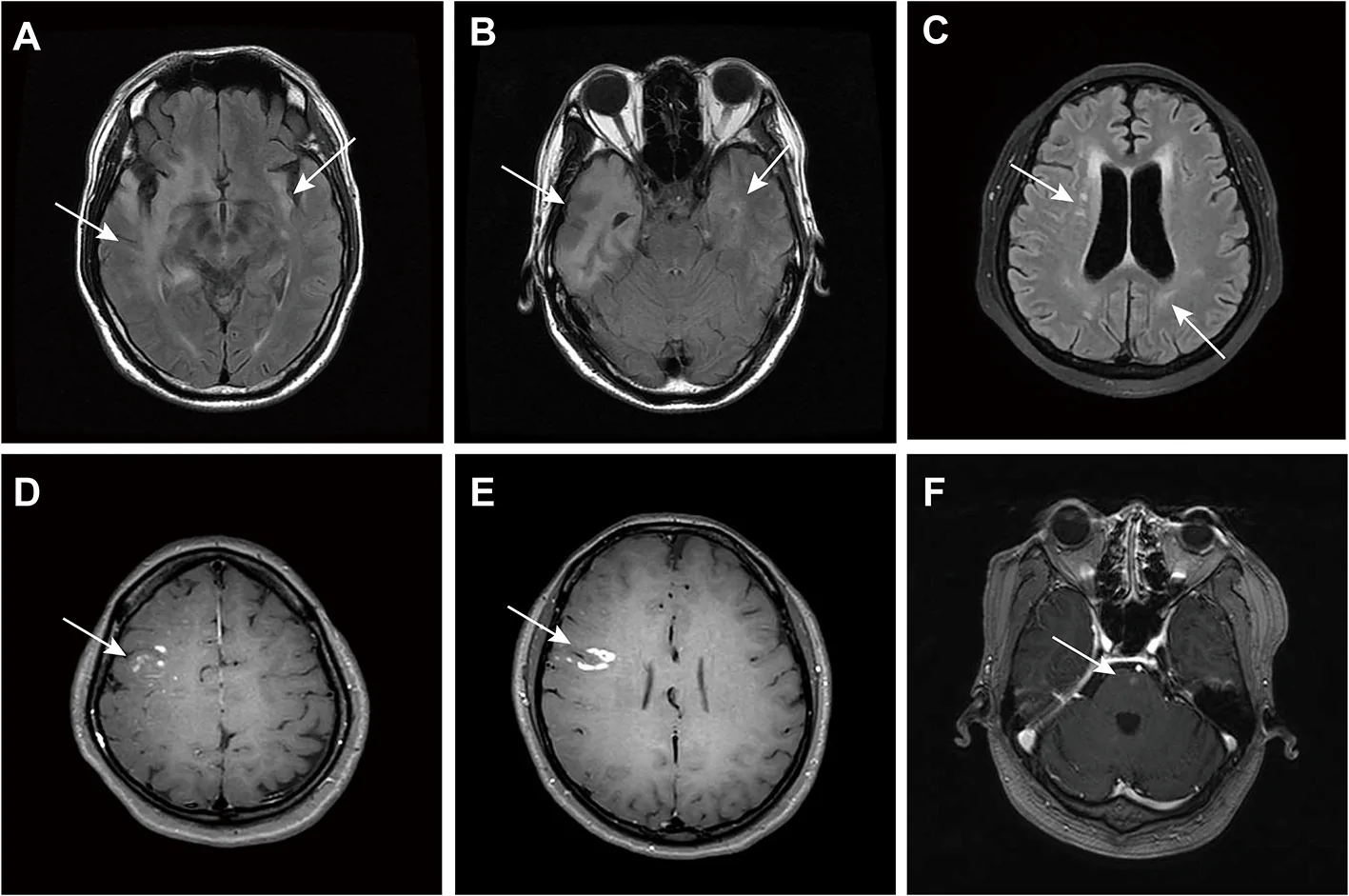
Brain magnetic resonance imaging (MRI) findings of patients with AE. **(A, B)** Plain brain MRI shows swelling of the bilateral temporal and insular lobes with patchy FLAIR hyperintensity (Case 4). **(C)** Plain brain MRI shows spotty FLAIR hyperintensity in the right frontal lobe and corona radiata (Case 3). **(D, E)** Plain and contrast-enhanced brain MRI show spotty abnormal enhancement with ill-defined margins in the right parietal and insular lobe parenchyma and parts of the adjacent sulci (Case 7). **(F)** Plain and contrast-enhanced brain MRI show a small nodular enhancement in the anterior part of the left pons (Case 8).

#### 3.2.5 Electroencephalogram

All 14 AE patients underwent EEG examination. Abnormal EEG findings were found in 10 patients (71.43%). Among them, nine patients (64.29%) showed increased slow waves, and two patients (14.29%) showed epileptiform discharges, predominantly in the frontal, temporal, and sphenoidal regions (**Table 3**).

### 3.3 Efficacy and prognosis of EFG treatment

#### 3.3.1 Assessment of the improvement of clinical symptoms

The median time from the onset of disease to the administration of EFG was 19.00 (12.75, 35.75) days. The frequency of EFG administration ranged from one to seven cycles, with a median of 2.5 cycles. Except for one patient who refused further treatment, treatment was discontinued in all other patients due to clinical improvement. After EFG treatment, 92.86% (13/14) of patients showed substantial clinical improvement, and the mean time to symptom improvement was 9.62 ± 8.18 days after treatment. Psychiatric symptoms were the earliest to improve in five patients (5/14). Additionally, one patient voluntarily discontinued treatment and was discharged 12 days after hospitalization. At the 12-week follow-up, the six patients who suffered seizures had reduced seizure frequency, and their symptoms were under control; seven patients had residual mild cognitive impairment, all manifesting as a mild reduction in memory (**Table 2**).

The mRS, CASE, GCS, and CGI scores were assessed in the 14 patients before and after EFG treatment (**Table 4**) to evaluate the overall or specific symptom severity and improvement. The baseline scores before treatment were as follows: mRS: 3.00 (3.00, 4.00), CASE: 5.00 (4.25, 12.75), and GCS: 14.50 (10.50, 15.00) (**Table 4**).

**Table 4:** Changes in mRS, CASE, NEOS, and GCS scores before and after EFG therapy [M(P25, P75)].

Score	Before EFG	1W	2W	3W	4W	12W
mRS	3.00 (3.00,4.00)	3.00 (2.00,3.75)	2.00 (1.00,3.00)	2.00 (1.00,3.00)	1.50 (1.00,2.00)	1.00 (1.00,1.00)
CASE	5.00 (4.25,12.75)	5.00 (2.50,8.25)	3.50 (2.25,5.75)	2.50 (2.00,4.00)	2.00 (1.25,2.00)	1.00 (1.00,1.75)
NEOS	1.00 (0.25,1.00)	NA	NA	NA	NA	0.00 (0.00,0.00)
GCS	14.50 (10.50,15.00)	NA	NA	NA	NA	15.00 (15.00,15.00)

After treatment with EFG (10 mg/kg/week), the mRS scores in weeks 1, 2, 3, 4, and 12 are shown in **Figure 2.1**. In week 1 after treatment, the proportion of patients with mRS ≤2 was 35.71%; in week 12, this proportion increased to 92.86%. The mRS scores decreased gradually over the follow-up period (**Figure 2.2**). Except for one patient who refused further treatment, the functional outcome for the remaining 13 patients was favorable. The CASE scores before and at one, two, three, four, and 12 weeks after EFG treatment (10 mg/kg/week) are shown in **Figure 3**. Baseline CASE scores ranged from 2 to 22. The CASE scores decreased during treatment, dropping to 1.00 (1.00, 1.75) 12 weeks after EFG treatment, indicating substantial improvement.

**Figure 2:**
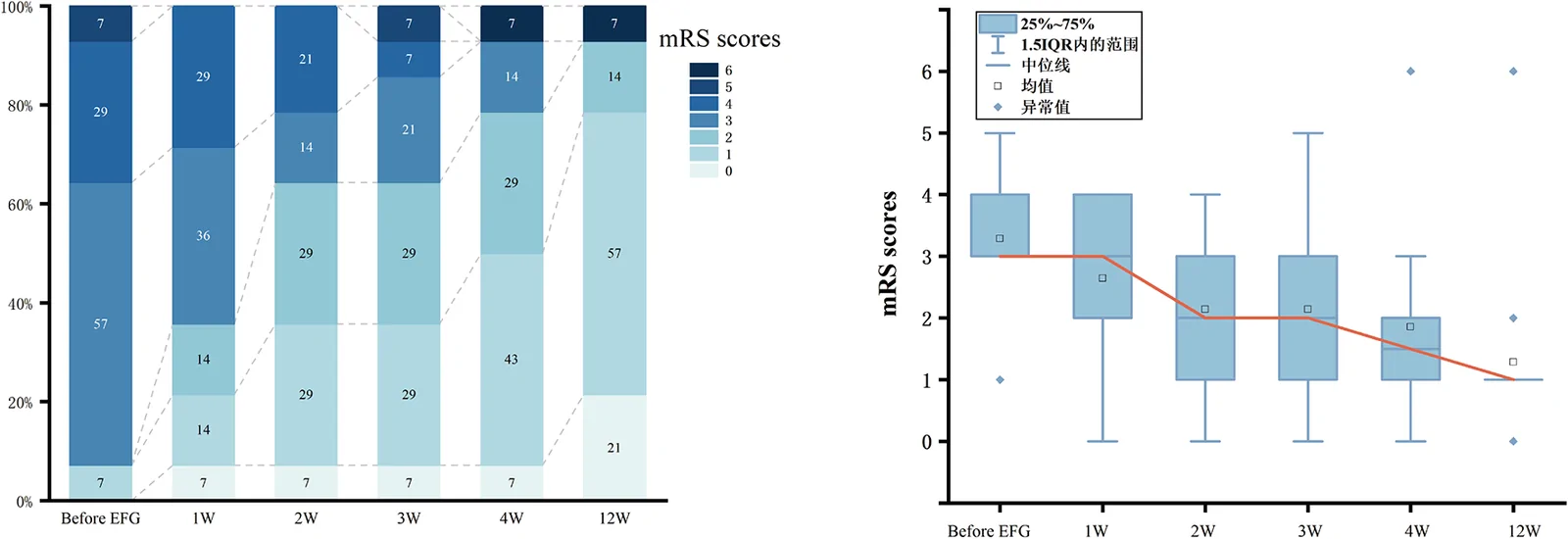
The mRS scores at baseline and follow-up time points.

**Figure 3:**
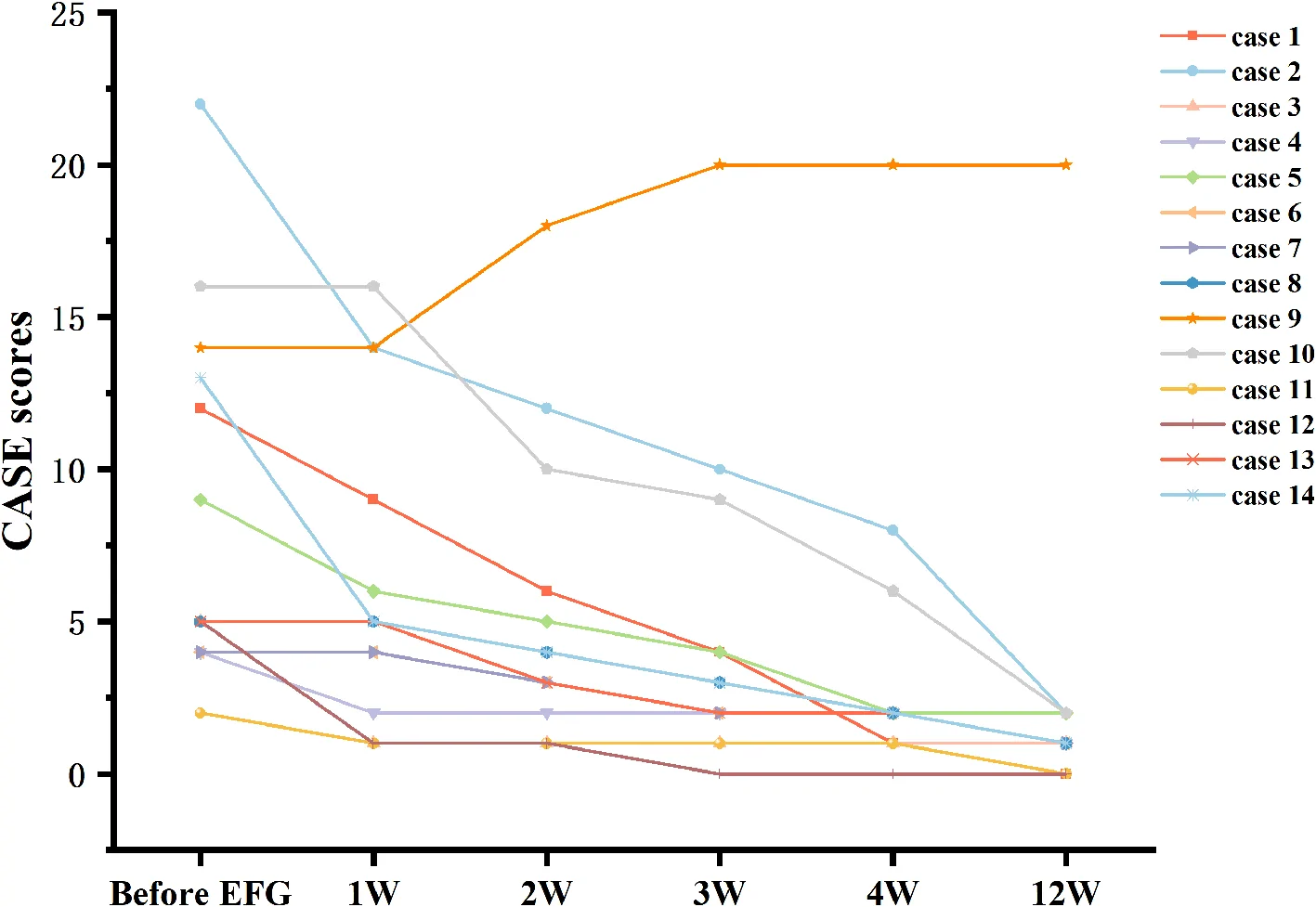
CASE scores at baseline and follow-up time points.

The Friedman test was conducted to analyze the overall changes in mRS and CASE scores across six time points: baseline and one, two, three, four, and 12 weeks after treatment. The results showed highly significant differences in the distribution of scores across the time points (**Table 5**). To identify the time point at which improvement occurred, the Wilcoxon signed-rank test was conducted to compare each follow-up time point with baseline. After implementing Bonferroni correction, the results showed that the mRS and CASE scores at two, three, four, and 12 weeks after treatment were significantly lower than the corresponding baseline scores. However, the differences in the mRS and CASE scores were not significant between week 1 and baseline (**Table 6**), which was consistent with the mean time to symptom improvement.

**Table 5:** Multiple comparisons of mRS, CASE scores, and serum total IgG levels across time points before and after EFG therapy (Friedman test).

Sources of variation	n	*X^2^*	df	*P*
mRS	14	38.288	5	<.001*
CASE	14	45.986	5	<.001*
Serum IgG	9	22.60	3	<.001*

*P< 0.05 indicates statistical significance.

**Table 6:** Wilcoxon signed-rank test of changes in mRS, CASE scores, and serum total IgG from baseline after EFG therapy.

Sources of variation	Statistic	1W *VS* Before EFG	2W *VS* Before EFG	3W *VS* Before EFG	4W *VS* Before EFG	12W *VS* Before EFG
mRS	Z	-2.264	-2.859	-2.818	-2.726	-2.969
	*P*	0.024*	0.004*	0.005*	0.006*	0.003*
CASE	Z	-2.530	-2.777	-2.675	-2.735	-2.737
	*P*	0.011*	0.005*	0.007*	0.006*	0.006*
Serum IgG	Z	-2.666	NA	-2.666	NA	-1.955
	*P*	0.008**	NA	0.008**	NA	0.051**

*P < 0.01 after Bonferroni correction indicates statistical significance.

**P < 0.0167 after Bonferroni correction indicates statistical significance.

Clinical Global Impressions (CGI) scores at one, two, three, four, and 12 weeks after EFG treatment (10 mg/kg/week) are shown in **Figure 4**. The CGI-Severity of Illness (CGI-SI) assessment revealed that the overall disease severity at one week after treatment was 3.64 ± 1.67, and it decreased during follow-up, dropping to 1.00 (1.00, 1.75) at 12 weeks after treatment, indicating that the disease was gradually improving. Efficacy assessment showed that 92.86% of patients achieved clinical improvement at the last follow-up; among them, 85.71% achieved marked improvement (clinical improvement was defined as CGI-Global Improvement [CGI-GI] ≤3, marked improvement was defined as CGI-GI ≤1). The clinical condition worsened in only one patient. The Efficacy Index (EI = efficacy evaluation/side effects) showed a median value of ≥2 throughout follow-up, indicating high efficacy with manageable side effects, representing an ideal state.

**Figure 4:**
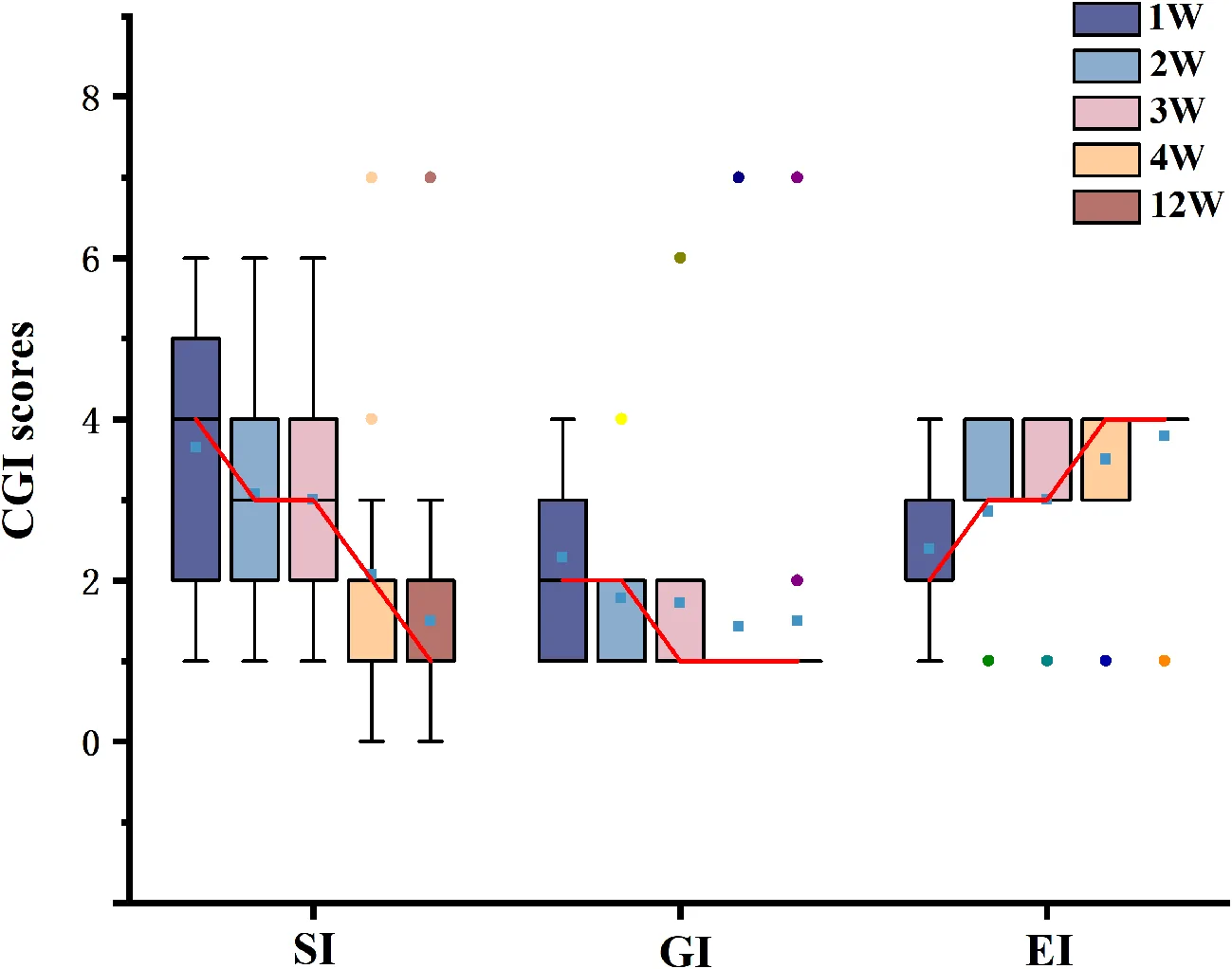
CGI at follow-up time points.

#### 3.3.2 Dynamic monitoring of total IgG in serum

Nine patients were regularly monitored for their total IgG levels in peripheral blood. The total IgG levels were evaluated before treatment and at one, three, and 12 weeks after treatment with EFG. The median baseline IgG level was 12.60 (11.9, 16.20) g/L; three patients had levels considerably above the upper limit of the normal range, with the highest level being 31.1 g/L. At one and three weeks after EFG treatment, total IgG levels in peripheral blood were considerably lower than baseline levels: at one week, the level dropped to 6.81 [5.24; 7.74] g/L; at three weeks, it dropped to 5.21 (4.32, 8.36) g/L (**Figure 5**). The Friedman test revealed significant differences in the total IgG levels in peripheral blood across the three time points (weeks 0, 1, and 3). The Wilcoxon signed-rank test with Bonferroni correction showed that the total IgG levels in peripheral blood at one week and three weeks after EFG treatment were significantly different from baseline (**Tables 5**, **6**). At 12 weeks, the median IgG level was 10.70 [7.90; 11.30] g/L, which was higher than the median IgG level at three weeks; the difference from baseline was not significant, but the level remained below baseline.

**Figure 5:**
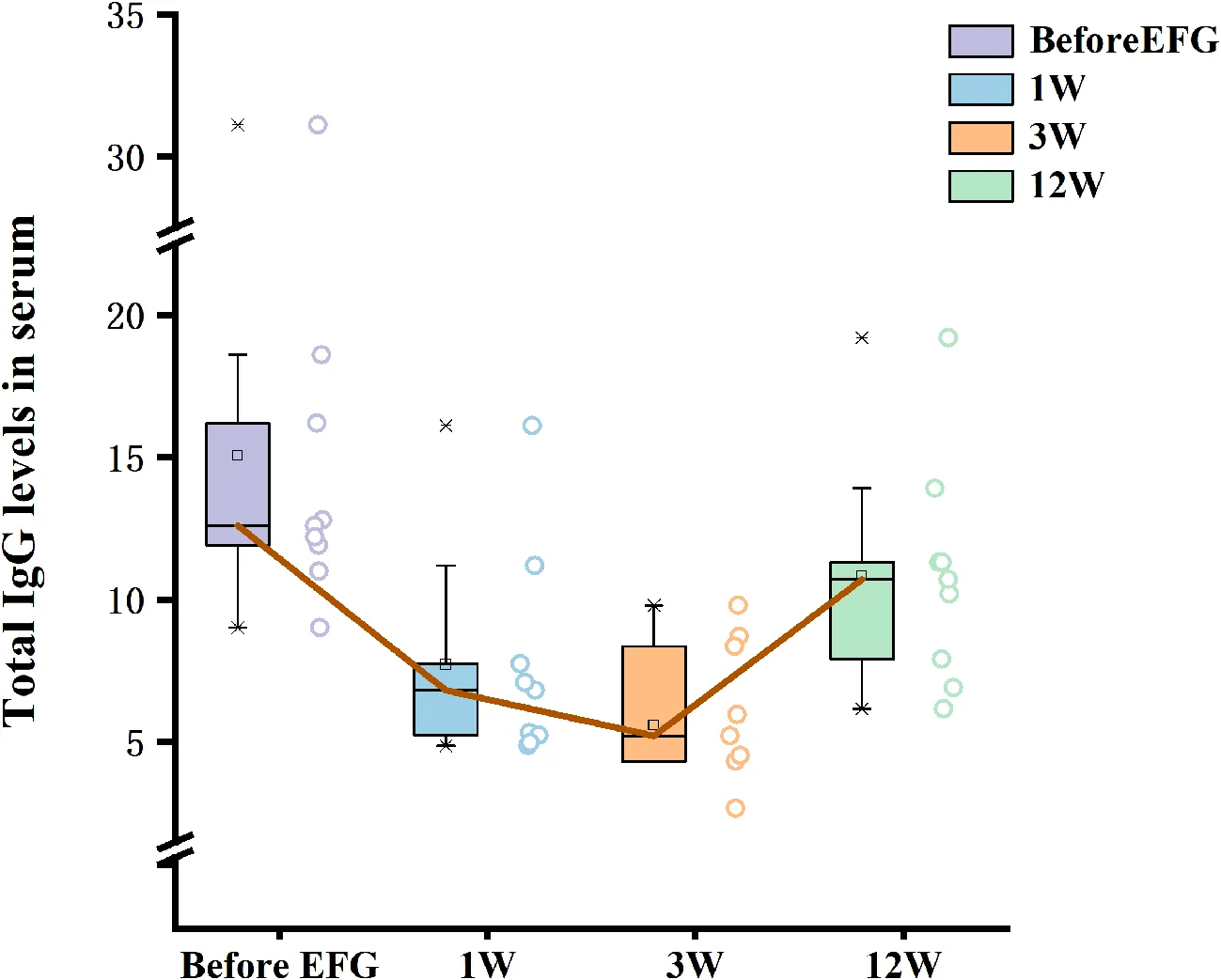
Total IgG levels in serum at baseline and follow-up time points.

#### 3.3.3 Prognosis assessment

At the last follow-up after EFG treatment, except for one patient who refused further treatment, the remaining 13 patients could care for themselves without assistance, and 11 of those patients had returned to normal life (mRS ≤1). At the last follow-up, the median mRS and CASE scores were both 1, and the score was 0 on both scales for three patients. The NEOS score, which was assessed before EFG treatment to predict prognosis, was 1.00 (0.25, 1.00) and decreased to 0.00 (0.00, 0.00) at the last follow-up, which indicated that the overall prognosis was favorable (**Table 4**). No relapses occurred during follow-up.

### 3.4 Adverse reactions and safety of EFG

Throughout the treatment period, adverse events were observed in four of the 14 patients. Detailed information regarding patient ID, event type, onset time, clinical course, and causal assessment is summarized as follows:

Three patients presented with abnormal clinical manifestations prior to EFG initiation, which were judged to be unrelated to EFG treatment. Patient 1 had pre-existing pulmonary infection and liver function impairment before EFG administration, which were considered secondary to long-term bedridden status and long-term antiepileptic drug use. Patient 2 developed drug rash before EFG treatment, which was attributed to prior antibiotic application. Patient 3 exhibited liver function impairment at baseline, which was associated with antiepileptic drugs and did not deteriorate after EFG initiation.

Only one adverse event was considered possibly related to EFG treatment. Patient 14 received EFG on the second day after admission and developed pulmonary infection on the fourth day of hospitalization. This infection was judged to be possibly associated with EFG administration. After one week of targeted antibiotic therapy, the pulmonary infection was significantly improved, and EFG treatment was continued without interruption.

No additional adverse reactions were recorded in the remaining patients. No severe AEs, such as permanent disability or death, were observed in any of the participants.

## 4 Discussion

Autoimmune encephalitis (AE) is a class of immune-mediated disorders of the CNS driven by autoantibodies, characterized by the host immune system attacking self-antigens expressed in the CNS. AE frequently involves the limbic system and cerebral cortex, with clinical manifestations including psychiatric and behavioral abnormalities, seizures, cognitive dysfunction, a reduction in consciousness levels, and movement disorders, which can lead to significant disability and even death (2).

With the development of anti-neuronal surface antibody detection techniques in recent years, the diagnostic rate of AE has increased considerably (14); however, AE treatment is still quite challenging. Most therapeutic evidence comes from retrospective data, with a lack of randomized controlled trials. The greatest evidence-based treatment experience comes from a large partially prospective cohort study in which treatment data on anti-NMDAR encephalitis were published (32). For other subtypes of AE, evidence is mainly based on smaller cohort studies or case series (33–35). Although conventional immunotherapies, such as GC, IVIG, and PE, are effective for most patients, some patients experience rapid disease progression or poor response, particularly prominent in relapsed, refractory, and severe cases. Moreover, the efficacy of IVIG may be influenced by the VNTR genotype of the patient (36), leading to large interindividual variability in response; some patients require higher doses or more frequent administration. Additionally, IVIG has a slow onset of action; clinical improvement often takes around two weeks (37), and treatment may not significantly reduce pathogenic antibody titers. Additionally, IVIG is expensive, and its supply depends on limited blood resources, which may restrict its use. GC is widely used as a first-line therapy, but its immunosuppressive effects are non-specific, and the side effects associated with long-term use, such as metabolic disturbances and a high risk of infection, are significant (38). For severe or refractory patients, PE or immunoadsorption is often selected as an intensified treatment. However, both procedures are invasive, usually requiring central venous catheterization. The treatment is time-consuming and is performed in the hospital setting (39). PE also depends on the supply of blood products and carries risks such as plasma allergy, deep vein thrombosis, and cross-infection; moreover, it can decrease the concentration of drugs in the blood. Although immunoadsorption may be more targeted in removing specific antibodies, its operational complexity limits its clinical convenience and widespread use. Therefore, novel therapeutic approaches with greater targeting, faster onset of action, and higher safety need to be developed. FcRn antagonists, along with other monoclonal antibodies (e.g., tocilizumab), are under evaluation as potential third-line therapeutic interventions for AE (40).

FcRn antagonists are a novel class of targeted immunomodulators. They have a precise, rapid, and safe mechanism of action; by binding to FcRn and inhibiting its function, they block the FcRn-mediated intracellular IgG recycling pathway, promoting the degradation and clearance of IgG, thereby reducing circulating levels of total IgG and pathogenic IgG autoantibodies without affecting the levels of other immunoglobulins (IgA, IgD, IgE, or IgM) (41). Their efficacy does not depend on blood products, and their route of administration is convenient (subcutaneous/intravenous), avoiding the risks and resource limitations of traditional invasive procedures (e.g., PE). Moreover, their selective mechanism of action keeps the risk of infection under control. Despite these advantages, several limitations of EFG itself should be acknowledged. The high cost of EFG may restrict its accessibility in clinical practice, particularly in resource-limited settings. Moreover, its long-term safety profile in AE patients has not yet been established, and optimal treatment protocols—including dosing, cycle length, and maintenance strategies—require further investigation. Additionally, the efficacy of EFG in antibody-negative AE or AE mediated by non-IgG mechanisms remains theoretically uncertain, as its mechanism relies on IgG clearance. This drug has shown favorable efficacy and safety in generalized MG and CIDP (42). Exploratory studies have also investigated the use of FcRn antagonists in other neurological diseases such as NMOSD, GBS, and anti-NMDAR encephalitis (28, 43, 44). However, only a few real-world studies have investigated the effectiveness of FcRn antagonists in the treatment of AE. In this study, the efficacy and safety of the FcRn antagonist EFG in treating AE in a real-world setting were retrospectively analyzed to provide clinical evidence for its use in AE. The results showed that after EFG treatment, most patients in this cohort exhibited rapid, significant, and sustained neurological improvement. By the follow-up in week 12, 92.86% of patients achieved a favorable functional outcome (mRS ≤2), with an overall good prognosis and good tolerability to treatment. This preliminary real-world evidence suggests that FcRn antagonists may be an effective and promising novel targeted therapy for refractory, relapsed, or severe AE and also provides valuable data for a deeper understanding of FcRn-targeted therapy in complex immune diseases of the CNS.

In this study, 14 patients with a confirmed diagnosis of AE who were treated with the FcRn antagonist EFG at the Second Hospital of Hebei Medical University between November 2023 and November 2025 were included. Among them, five patients (35.71%) met the definition of refractory AE, two patients (14.29%) were relapsed cases, and five patients (35.71%) were severely ill at baseline (mRS ≥4); thus, the overall disease burden in this cohort was high. Most patients in this study had already received multiple immunotherapeutic interventions. All patients had received at least one first-line immunotherapy (GC and/or IVIG/PE), and some had even progressed to more advanced immunosuppressive stages, including 28.57% who received OFA (a B-cell-targeting CD20 monoclonal antibody), 14.29% who received RTX, and 35.71% who received MMF for maintenance therapy. This indicates that the spectrum of AE patients in real clinical practice is complex. With this as the background, FcRn antagonist therapy was introduced to intervene at a more upstream step of “antibody clearance.” The results showed that the symptoms improved significantly in 92.86% of patients after EFG treatment, with the mRS, CASE, and CGI scores showing sustained reductions. At the 12-week follow-up, 13 patients had mRS ≤2; among them, 11 patients returned to normal life, which indicated that they achieved a favorable functional outcome. Additionally, seizures were under control, and psychiatric symptoms, motor function, and impaired consciousness improved to different degrees. The overall clinical efficacy was high, which was particularly valuable given the high proportion of relapsed, refractory, or severe cases.

This study found that the FcRn antagonist EFG has a rapid onset of action. The mean time to symptom improvement was 9.62 days after treatment. Compared to the response times reported by Yao Tan et al. (42) for PE (14 days), repeated IVIG (19 days), CTX (22 days, 47.1% response), and RTX (15 days), EFG has a significant advantage. Moreover, the mRS and CASE scores started decreasing significantly from the second week after treatment, whereas the changes in week 1 were not significant; this finding was consistent with the mean time to symptom improvement (9.62 days) observed in this study. This study also adopted a composite assessment system that included the overall degree of neurological dysfunction and disability (mRS), disease-specific severity (CASE), severity of acute impaired consciousness (GCS), and global clinical impression (CGI), cross-validating the efficacy from different dimensions. The CGI assessment showed that most patients experienced clinically meaningful global improvement following EFG treatment, and the EI also indicated that the treatment was in the ideal range of “marked efficacy with manageable side effects”. The reduction in CGI-SI scores was consistent with the improvement in CGI-GI scores and also consistent with the improvement in the mRS and CASE scores, jointly confirming that the treatment was effective. This multi-tool, multi-dimensional consistency greatly increases the reliability of our conclusions. Analysis of the improvement of specific symptoms revealed that psychiatric symptoms, movement disorders, and impaired consciousness improved the earliest. All six patients with seizures had lower seizure frequency after treatment, confirming that FcRn antagonists can improve the core symptom cluster of AE. These observations may help clinicians more accurately predict which symptoms are most likely to respond rapidly to FcRn antagonist therapy, helping them optimize patient management.

The core immunopathological mechanism underlying the effects of AE lies in pathogenic autoantibodies, particularly IgG antibodies against neuronal cell membrane receptors or synaptic proteins. This study not only included the most common anti-NMDAR encephalitis (11 cases) but also covered rare types such as anti-LGI1, anti-mGluR5, and anti-IgLON5 antibody-associated encephalopathy; all patients benefited from the treatment except for one patient with anti-NMDAR encephalitis, who failed to improve owing to refusal of further EFG treatment. Restricted by the small sample size, we did not perform formal statistical subgroup analyses; therefore, we cannot draw definitive conclusions regarding which subgroup of AE patients is more sensitive to EFG treatment. Further validation via larger-sample multicenter cohort studies is needed. This finding clinically confirms that FcRn serves as a regulator of the IgG recycling pathway, and its antagonists have potential efficacy in all IgG-mediated subtypes of AE. Although the pathogenic mechanisms mediated by different antibodies (e.g., receptor cross-linking and internalization, blocking receptor function, and interfering with protein-protein interactions (45)) have differences, the final common pathway involves the presence of pathogenic IgG. Therefore, clearance of pathogenic IgG is likely to yield good therapeutic outcomes. Nine patients in this study were monitored for total IgG levels in peripheral blood. The results showed that total IgG levels decreased significantly at one week and three weeks after treatment compared to baseline. At one week after treatment, the median IgG level dropped sharply by ~46%, from a baseline of 12.60 g/L to 6.81 g/L. This rapid reduction in peripheral blood IgG levels was temporally synchronized with the early improvement in clinical scores, suggesting that the decrease in total IgG levels in peripheral blood may be correlated with clinical efficacy. This supports the core mechanism that “FcRn antagonists accelerate the systemic clearance of IgG, including pathogenic autoantibodies, thereby rapidly and effectively reducing their attack on the CNS and mediating the improvement of clinical symptoms” (28, 46, 47). At the 12-week follow-up, the level had rebounded (10.70 g/L) but remained below baseline, whereas clinical efficacy had stabilized without relapse. This finding suggests that the therapeutic effect of FcRn antagonists may not completely depend on sustained deep suppression of IgG. However, existing studies cannot fully elucidate the relationship between IgG changes and long-term prognosis. Continuous monitoring of the titers of specific pathogenic antibodies (e.g., anti-NMDAR antibodies) in CSF and serum will be necessary to more precisely establish the causal relationship between antibody clearance and clinical outcomes.

Safety of novel immunotherapeutic techniques is an important prerequisite for their widespread application. During the follow-up period, no SAEs such as severe disability or death were observed in this study. Other studies have also suggested that FcRn antagonists have a generally favorable safety profile, with the main adverse reactions including respiratory or urinary tract infections or headache (41). The findings of this study are largely consistent with these reports, but it is still necessary to improve the monitoring of infections, pay attention to changes in liver function and hematological parameters, and perform risk assessment and dynamic management if required in clinical practice.

Beyond the efficacy and safety data discussed above, our findings raise an important clinical question: could efgartigimod (EFG) be incorporated into the earlier stages of autoimmune encephalitis (AE) treatment, rather than being reserved solely for salvage therapy after conventional treatment failure? In this cohort, all patients received EFG only after showing an inadequate response to first-line and/or second-line immunotherapies (median time from disease onset to EFG administration: 19.00 days; IQR: 12.75–35.75 days). Therefore, our data do not permit a direct conclusion that EFG should replace current standard first-line therapies. Nevertheless, several observations provide indirect support for earlier intervention. In our cohort, EFG demonstrated a rapid onset of action (mean 9.62 days), which compares favorably with reported response times for conventional therapies (as detailed in the preceding paragraphs). Even with a high proportion of refractory (35.71%), relapsed (14.29%), and severe cases (35.71% with baseline mRS ≥4), 92.86% of patients achieved favorable functional outcomes (mRS ≤2) by week 12. These observations, combined with EFG’s targeted mechanism of action and favorable safety profile, suggest that EFG holds promise as an earlier therapeutic option. However, we acknowledge that this hypothesis requires confirmation through prospective randomized controlled trials directly comparing first-line EFG with standard immunotherapies.

Although the sample size of this study was small, it covered multiple types of antibodies and had strong real-world attributes, with complex baseline patient conditions and diverse treatment modalities, making it closer to clinical practice. Moreover, the degree of functional recovery, time to recover, and clinical remission rate in this study were better than those reported for some traditional immunotherapeutic strategies (42), suggesting that the strategy implemented in this study can shorten the disease recovery cycle and improve the quality of life of patients. This provides a new targeted treatment option for AE patients who respond poorly or are intolerant to first-line and/or second-line immunotherapeutic agents (e.g., IVIG, GC). However, this study had several limitations. First, as this was a single-center retrospective study with a small sample size of only 14 patients, it may have introduced selection bias and made it difficult to comprehensively represent the entire AE patient population. Second, longitudinal laboratory and follow-up data were incomplete within this cohort. Although we observed a temporal trend between rapid peripheral-blood total IgG reduction and early clinical improvement, serial total IgG monitoring was only available for nine patients. Therefore, formal statistical correlation analyses between the magnitude of IgG reduction and the degree of clinical symptom improvement could not be performed. Third, the lack of systematic dynamic monitoring of pathogenic antibody titers in CSF and serum in all patients hinders mechanistic correlation analysis. Fourth, our retrospective analysis could not determine the optimal concentration, therapeutic cycle, or administration timing of EFG in AE. We did not perform therapeutic drug monitoring (e.g., peak or trough EFG concentrations), as this is not part of routine clinical practice in our center. Although the observed reduction in total IgG levels provided indirect pharmacodynamic evidence supporting the standard 10 mg/kg weekly regimen, the optimal cycle length likely depends on individual patient characteristics (including baseline severity, antibody type and titer, and response kinetics), and the optimal administration timing for EFG in the AE treatment algorithm remains unknown. Future prospective dose-finding and pharmacokinetic-pharmacodynamic studies are warranted to optimize these parameters. Additionally, the follow-up duration of this study was relatively short (12 weeks), precluding assessment of the long-term efficacy, optimal maintenance treatment regimen, and long-term relapse risk of EFG. Finally, because some patients had received other immunosuppressive treatments and a randomized control group was lacking. Although no new adjunctive immunotherapies were administered after EFG initiation, the contribution of sustained or delayed effects of concomitant treatments could not be completely excluded; therefore, the evidence is relatively weak.

In the future, multicenter, large-sample, prospective randomized controlled trials are needed to provide high-level evidence, validate the efficacy and safety of FcRn antagonists in AE, and evaluate their application value in patients with different types of antibodies, disease stages, and severity levels. Moreover, the optimal treatment window (whether as a component of first-line acute therapy or only for refractory/relapsed cases), dosing regimen and course, and the effect of combination with conventional immunotherapy should be further investigated. Additionally, systematic monitoring of total IgG and specific pathogenic antibody titers in both cerebrospinal fluid (CSF) and serum, together with other immunological parameters including CSF protein electrophoresis and T-cell immunophenotyping, is required to establish a more precise mechanistic correlation between antibody clearance and clinical outcomes, as well as conducting long-term follow-up studies to evaluate long-term safety, long-term effects on cognitive function, and relapse rates, may improve the precision and facilitate individualized development of treatment for AE.

## 5 Conclusion

This retrospective study involving 14 patients with AE showed that the FcRn antagonist EFG has favorable clinical efficacy in AE. For patients who responded poorly to first-line and/or second-line immunotherapy, particularly those with refractory or severe AE, FcRn antagonist therapy induced rapid, significant, and sustained improvement in clinical symptoms and neurological function, helped most patients achieve a favorable functional outcome, and exhibited an overall good safety profile with manageable adverse reactions. The rapid reduction in the total IgG levels in peripheral blood, which occurred along with clinical improvement, provides strong supportive evidence for its mechanism of action. Although validation through higher-quality randomized controlled trials is needed, this study provides preliminary real-world data for the clinical application of EFG in AE treatment and offers clinicians a novel targeted treatment option, carrying exploratory significance.

## Data Availability

The raw data supporting the conclusions of this article will be made available by the authors, without undue reservation.
