# Supplementary material for: Real-world efficacy and safety of efgartigimod in patients with autoimmune encephalitis: a single-center retrospective analysiss
Source: Front Immunol. 2026 Oct 8;17:1963052. doi: 10.3389/fimmu.2026.1963052 (PMC13687301; doi:10.3389/fimmu.2026.1963052)
Supplement: Supplementary file 1 [file Table1.docx]

Table S1 Detailed immunotherapy history of 14 patients with AE before EFG initiation

| Patient ID | Antibody subtype | Disease phenotype  (firs-onset/refractory/relapsed) | Prior immunotherapies before EFG |
| --- | --- | --- | --- |
| Case 1 | Anti-NMDAR antibody | Refractory | IVIG, GC, PE, OFA, MMF |
| Case 2 | Anti-NMDAR antibody | Refractory | IVIG, GC |
| Case 3 | Anti-NMDAR antibody | Relapsing | IVIG, GC, OFA, MMF, RTX |
| Case 4 | Anti-NMDAR antibody | Initial onset | IVIG, GC, MMF |
| Case 5 | Anti-NMDAR antibody | Initial onset | IVIG, GC |
| Case 6 | Anti-NMDAR antibody | Relapsing | IVIG, GC |
| Case 7 | Anti-NMDAR antibody | Initial onset | GC |
| Case 8 | Anti-NMDAR antibody | Relapsing | IVIG, GC, OFA, MMF, RTX |
| Case 9 | Anti-NMDAR antibody | Initial onset | IVIG, GC |
| Case 10 | Anti-NMDAR antibody | Initial onset | GC, OFA |
| Case 11 | Anti-NMDAR antibody | Initial onset | IVIG, GC, MMF |
| Case 12 | Anti-LGI1 antibody | Relapsing | IVIG, GC |
| Case 13 | Anti-mGluR5 antibody | Relapsing | GC |
| Case 14 | Anti-IgLON5 antibody | Initial onset | IVIG |
